# Multicenter Prospective Clinical Study on Chairside‐Fabricated Partial Crowns: 5‐Year Results for Lithia‐Zirconia Glass–Ceramic Restorations

**DOI:** 10.1111/jerd.13328

**Published:** 2024-10-09

**Authors:** Sven Rinke, Emanuel Reinermann, Andreas Leha, Matthias Roediger, Dirk Ziebolz

**Affiliations:** ^1^ Department of Prosthodontics University Medical Center Goettingen Germany; ^2^ Department of Medical Statistics University Medical Center Goettingen Germany; ^3^ Department of Cariology, Endodontology and Periodontology University Medical Center Leipzig Germany

**Keywords:** chairside, clinical evaluation, lithia‐zirconia glass–ceramic (LZGC), partial crown, success rate, survival rate

## Abstract

**Objectives:**

Clinical evaluation of chairside‐fabricated lithia‐zirconia glass–ceramic (LZGC) partial crowns (CCPCs) in a multicenter practice‐based study.

**Materials and Methods:**

Seventy‐one patients were restored with 92 adhesively luted CCPCs (Cerec SW 4.2/Cerec MC XL/Celtra Duo) in three private dental clinics (C1–C3). Time‐dependent (Kaplan–Meier) survival rates (SVR) and success rates (SCR) were calculated. The following possible covariates of SVRs and SCRs were evaluated in a Cox regression model: Restoration position (premolar/molar), luting material (Variolink/Calibra), and operator (C1–C3).

**Results:**

Seventy‐three CCPCs were placed in 59 patients and were included in the study (mean observational period: 58.0 ± 15 months). Four complete failures (two tooth fractures, one restoration fracture, and one endodontic failure) were recorded. All failures and interventions occurred in one of the three centers (5‐year SCR: C1 + C2: 100%; C3: 71%; 95% confidence interval: [0.55; 0.87]). Additionally, three biological, and two technical complications required clinical intervention to maintain function, and all occurred in C3. Restorations placed in C1 and C2 showed a significantly reduced risk for failure/intervention (hazard ratio = 0.103, *p* = 0.026) compared with restorations placed in C3.

**Conclusions:**

LZGC CCPCs showed good five‐year clinical performance. However, SVRs and SCRs were significantly influenced by the operator. Additional clinical data are required for a more detailed investigation of this effect.

## 1 Introduction

Ceramic partial coverage restorations are a more conservative treatment option than full coverage crowns for the restoration of massively damaged posterior teeth. Apart from the traditional labside fabrication in at least two appointments, computer‐aided design and computer‐aided manufacturing (CAD/CAM) using intraoral scanners and chairside milling technology allow for the fabrication of partial coverage restorations in a single appointment (chairside‐fabricated ceramic partial crowns = CCPCs) [1, 2, 3].

The digital chairside workflow is mainly used for the fabrication of restorations for single teeth in various indications, including inlays, onlays, partial crowns, and full crowns [3]. Among the various dental materials available for CAD/CAM fabrication, dental ceramics have a long history in the chairside process [4, 5, 6]. Ceramics used for the CAD/CAM process should be mechanically strong and offer fast machinability [1, 3, 4, 5]. In the beginning, mainly feldspathic ceramics (e.g., Vita MKII, Vita Zahnfabrik H. Rauter GmbH and Co. KG, Bad Säckingen, Germany) were used for the production of chairside‐fabricated restorations [6]. These types of ceramics demonstrated good clinical performance for inlays [2, 7]. However, when used for posterior partial or full crowns, increased material fracture rates were detected [8, 9, 10, 11, 12, 13]. To reduce the risk of technical complications (i.e., material fractures) in CAD/CAM‐fabricated restorations, various particle‐filled glass–ceramics with improved mechanical properties (e.g., lithium disilicate ceramics (IPS.emax, Ivoclar Vivadent, Schaan, Liechtenstein)) have been adapted for use in the CAD/CAM fabrication process [3, 4, 5, 6]. For conventionally fabricated posterior partial and full coverage restorations made from this group of materials, high survival and success rates with reduced technical failure and complication rates were demonstrated in various clinical trials with observational periods of up to 16 years [14, 15, 16, 17].

For CAD/CAM‐fabricated partial and full coverage crowns, a reduced technical complication rate was also documented but only for limited observational periods of up to 5.5 years [18, 19]. These results verify that technical complications can be reduced by using restorative materials with improved strength (i.e., lithium disilicate ceramics). Further developments in the field of chairside‐fabricated restorations were aimed at the development of materials that provide not only improved mechanical strength (three‐point bending strength > 350 MPa) but also polishing characteristics, improved translucency, and reduced processing time [1, 5, 13]. One of the latest advancements with respect to chairside CAD/CAM ceramic materials is the development of lithia‐zirconia glass–ceramics (LZGCs) [1, 4]. As described by the manufacturers, these ceramics consist of 10% dissolved zirconium dioxide in a glassy matrix as well as fine lithium metasilicate, lithium orthophosphate, and lithium disilicate crystals (average size: 0.5–0.7 μm) [1, 6].

Since their introduction in 2013, LZGC ceramics have been investigated in a number of in vitro studies [20]. These studies revealed a characteristic strength that, when compared with silicate ceramics and zirconia, is found to be in the intermediate range. A comparable or slightly inferior fracture strength compared to lithium disilicate was reported [13, 21]. Nevertheless, LZGCs are reported to be a biocompatible material with good polishing characteristics and a high level of translucency, and their fracture resistance can withstand physiological chewing loads even at a reduced thickness of 1.0 mm [20]. The bonding characteristics of the LZGCs are similar to those of well‐established lithium disilicate materials [22, 23]. Although LZGC seems promising, especially for chairside fabrication, clinical data regarding these materials are still sparse. A recently published systematic review reported promising short‐term clinical outcomes (1‐year survival rate: 99%) for LZGC full‐ and partial coverage restorations. Nevertheless, studies with prolonged observational periods are needed to confirm the long‐term safety and performance of this group of materials [24].

In addition to the restorative material, various risk factors such as abutment type (premolar vs. molar), abutment vitality, cementation technique, or operator experience can affect the clinical long‐term reliability of indirectly fabricated restorations [7, 11, 15, 23, 25]. Nevertheless, no data on the clinical evaluation of these potential risk factors in LZGC CCPCs is available [24]. The current prospective practice‐based study seeks to investigate the clinical performance of CCPCs (Cerec system, Dentsply Sirona, Bensheim, Germany) constructed from completely crystallized LZGC (Celtra Duo, Dentsply Sirona, Bensheim, Germany) and the dependence of the outcome on the parameters luting technique, position of the CCPC (premolar vs. molar), as well as the clinical settings, including the operators' experience. A publication of the 3‐year results of this study is already available [26]. In the present study, the null hypothesis assumed that the clinical outcome (survival and success rates) does not depend on these risk factors: the position of the CCPCs, luting technique, and operator experience.

## 2 Materials and Methods

### 2.1 Patients

For the present study, 71 patients (45 female/26 male) were rehabilitated with complete cusp‐covering CCCPs made of a LZGC material. The details of the applied inclusion and exclusion criteria have already been published [26].

Written informed consent from every patient for participation in the study and approval of the study protocol by the responsible ethics committee (Georg‐August University, Goettingen, Germany (No. 10/4/13)) were obtained.

### 2.2 Clinical Treatment and Clinical Evaluation

Clinical treatment and clinical evaluation protocols were described in a previous publication [26]. Therefore, only a brief summary of the relevant clinical steps is given below.

#### 2.2.1 Clinical Treatment

**FIGURE 1:**
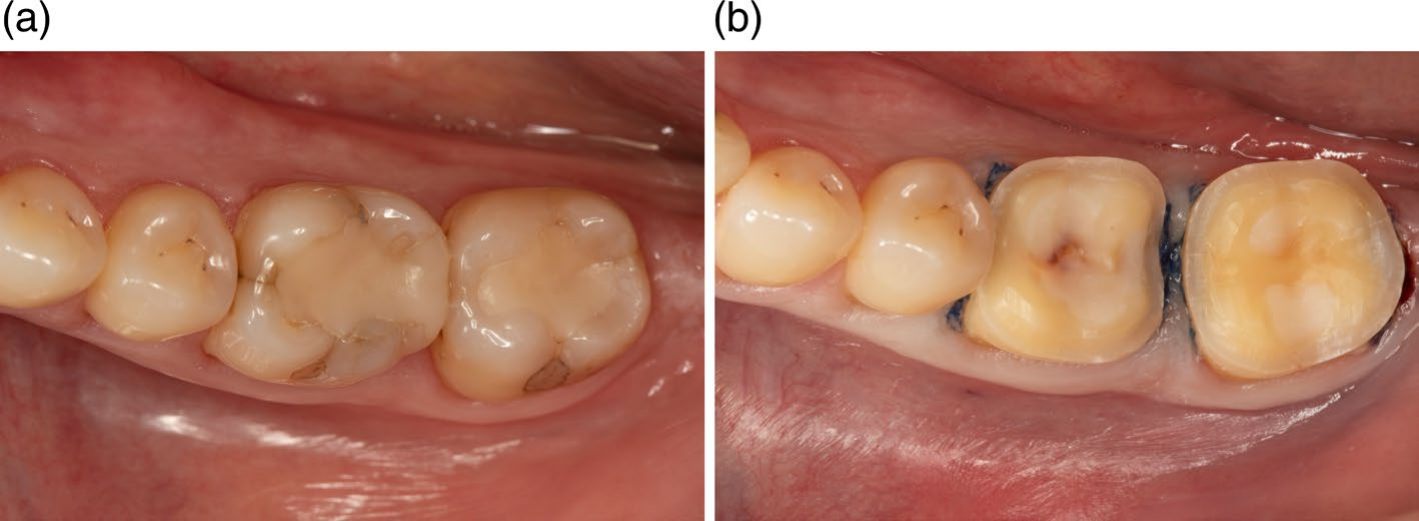
(a) Lower first and second molars with an indication of a CCPC were included in the present study. (b) Clinical situation after preparation for the CCPCs with complete cusp coverage.

All treatments were performed between October 01, 2013, and September 30, 2014, in three private practices by three dentists who had different levels of clinical experience with the Cerec system. Two dentists had more than 10 years of experience with various generations of the Cerec system (centers 1 and 2), whereas the third dentist (Center 3) had a three‐year history of working with the Cerec system. A preparation allowing a minimum material thickness of 1.5 mm for complete cusp coverage was established for CCPCs, (Figure 1a,b). Maxillary and mandibulary optical impressions (quadrant scans) plus lateral scans for bite registration were obtained with a powder‐free intraoral scanner (Cerec AC Omnicam, Dentsply Sirona, Bensheim, Germany). Afterward, a monolithic CCPC (Cerec software 4.2, Dentsply Sirona, Bensheim, Germany) was constructed with the design mode “Biogeneric Individual” [26]. The following restoration parameters were selected for all restorations: spacer occlusal, 120 μm; marginal adhesive gap, 60 μm.

After wet milling of the CCPCs using the milling mode “fine” (Cerec MCXL, Dentsply Sirona, Bensheim, Germany), the restorations were glazed and stain‐fired (maximum two firing cycles per restoration).

For adhesive cementation, the restorations were etched (5% hydrofluoric acid/30 s) and silanized. Adhesive cementation of the CCPCs was performed under a rubberdam using a total‐etch technique (30 s enamel, 15 s dentin) with one of these dual‐curing composite cement:

- Group A: Monobond S Plus, Syntac classic, and Variolink (VL) (Ivoclar Vivadent, Schaan, Liechtenstein).
- Group B: Calibra Cementation System (CCS), with the following components: Calibra Silane, XP Bond, Calibra Ceram (Dentsply Sirona, Bensheim, Germany).

Participants were assigned to the cementation groups according to the randomization schedule generated by an online statistical computing web program (www.randomization.com). Each restoration was polymerized for a total of 200 s (40 s each for the occlusal, mesial, distal, buccal, and lingual aspects). Dynamic occlusion was reconstructed to match the pretreatment clinical situation (i.e., unilateral dynamic guidance or anterior‐canine guided occlusion).

#### 2.2.2 Clinical Evaluation

**FIGURE 2:**
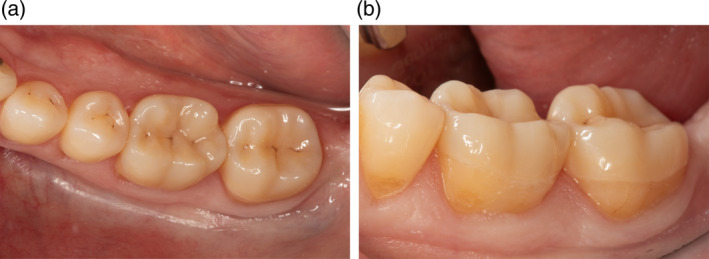
(a, b) CCPC restorations at baseline examination. The marginal adaptation was rated “alpha,” and the USHPS‐criteria marginal discoloration was rated “alpha” for both restorations.

**FIGURE 3:**
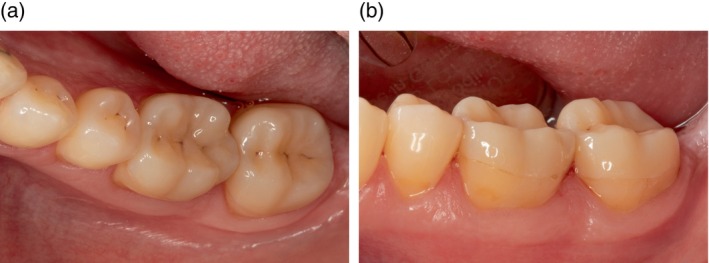
(a, b) CCPC restorations at 5‐year clinical follow‐up examination. The restorations remained intervention‐free in function. The USPHS criteria marginal adaptation and marginal discoloration were both rated “bravo.”

During the annual clinical follow‐up appointments, these parameters were evaluated: Survival rate (restoration remained in situ), success rate (intervention‐free functional period of the restoration), and modified USPHS criteria. Every restoration was examined regarding tooth vitality (CO_2_ testing), fissures, fractures, loosening, and caries. The following modified USPHS criteria were applied to rate the marginal adaptation and marginal discoloration [12] (Figures 2a,b and 3a,b):

**Table:** 

Marginal adaptation	alpha	Margin not discernible, probe does not catch
	bravo	Probe catches on margin but no gap, dentin, or liner exposed
	charlie	Probe catches on margin and gap on probing, dentin or liner exposed
	delta	Restoration fractured or missing
Marginal discoloration	alpha	No marginal discoloration
	bravo	Marginal discoloration, not penetrated toward pulp
	charlie	Marginal discoloration penetrated toward pulp

Between September 2019 and February 2020, a trained dentist (Emanuel Reinermann) conducted the last follow‐up examinations. This dentist did not participate in the treatment phase. Training on the evaluation parameters and clinical criteria was provided by one of the authors (Dirk Ziebolz) and was repeated until inter‐rater reliability achieved a substantial correlation, as indicated by Cohen's kappa (*k* ≥ 0.6) [26]. Negative events (restoration loss, ceramic fracture, biological complications, recementation if necessary) that led to failures occurring prior to examinations were documented in the patient files and considered in the final results.

### 2.3 Statistical Analysis

The survival and success rates of the reconstructions were the basis for statistical analysis. A restoration was classified as survival if it was still in place at the time of the follow‐up examination and showed no signs of complete failure (i.e., it met the in situ criterion) [26, 27]. A restoration was deemed a total failure if it experienced a clinically unacceptable ceramic fracture or if a biological issue (such as caries, tooth fracture, or periodontal disease) necessitated its complete replacement or the removal of the affected tooth. Reconstructions remaining unchanged and functional in situ without any intervention during the observational period were defined as success [26, 27]. Kaplan–Meier survival analysis was used to calculate the time‐dependent survival rates (based on the in situ criterion) and the success rates (intervention‐free).

The cementation material (VL vs. CCS), the position of the restoration (premolar vs. molar), and clinical settings, including the operators' experience (centers 1–3), were evaluated as potential covariates affecting the time‐dependent survival and success rates. Multiple observations from the same patient (several CCPCs per patient) were considered dependent, and variance estimation was adapted using a Cox regression model. Consequently, a marginal model was used for data analysis [28]. A *p* value of less than 0.05 was considered statistically significant. All analyses were conducted using the R statistical software (version 3.5.3; R Core Team 2018) with the “survival” package (version 2.44.1.1) and the “prodim” module for time‐to‐event analyses. The changes in the clinical criteria “marginal adaptation” and “marginal discoloration” from baseline to the 5‐year examination were evaluated separately for each luting procedure. A chi‐square test was used for the statistical analysis of the data (*α* = 0.05) [26].

## 3 Results

### 3.1 Study Population

Out of the 71 patients originally restored (45 female, 26 male; mean age 48.9 ± 12.9 years), 59 patients with 73 crowns attended regular annual follow‐up examinations, resulting in a 5‐year patient recall rate of 83% (Table 1). The 5‐year follow‐up examinations were conducted between September 2019 and February 2020 (observational period: 58.0 ± 15 months). Twelve patients (six female and six male, with a total of 19 restorations: One premolar and 18 molar) missed the final clinical examination, and their data were censored as of the date of their last available information. Documented reasons for not attending the final examinations included: Seven patients moved out of the area, two patients died, two patients suffered from serious illness, and one patient declined study participation. Thirty‐six restorations were assigned to Group A and 37 to Group B. Of the examined restorations, 31 were placed in the maxilla and 42 in the mandible (17 premolar and 56 molar partial crowns). Sixty‐eight of the restorations were placed on vital abutments, while five partial crowns were fitted on posterior teeth that had been properly endodontically treated.

**TABLE 1:** Distribution of the originally placed chairside‐fabricated ceramic partial crowns (CCPCs) and the drop‐outs registered in the participating three centers.


Patients and restorations (CCPC)			
	Center 1	Center 2	Center 3	Total
Baseline	17 patients/29 CCPCs	28 patients/30 CCPCs	26 patients/33 CCPCs	71 patients/92 CCPCs
Drop‐out	2 patients/4 CCPCs	3 patients/3 CCPCs	7 patients/12 CCPCs	12 patients/19 CCPCs
5‐year recall	15 patients/25 CCPCs	25 patients/27 CCPCs	19 patients/21 CCPCs	59 patients/73 CCPCs

### 3.2 Survival Rate

**FIGURE 4:**
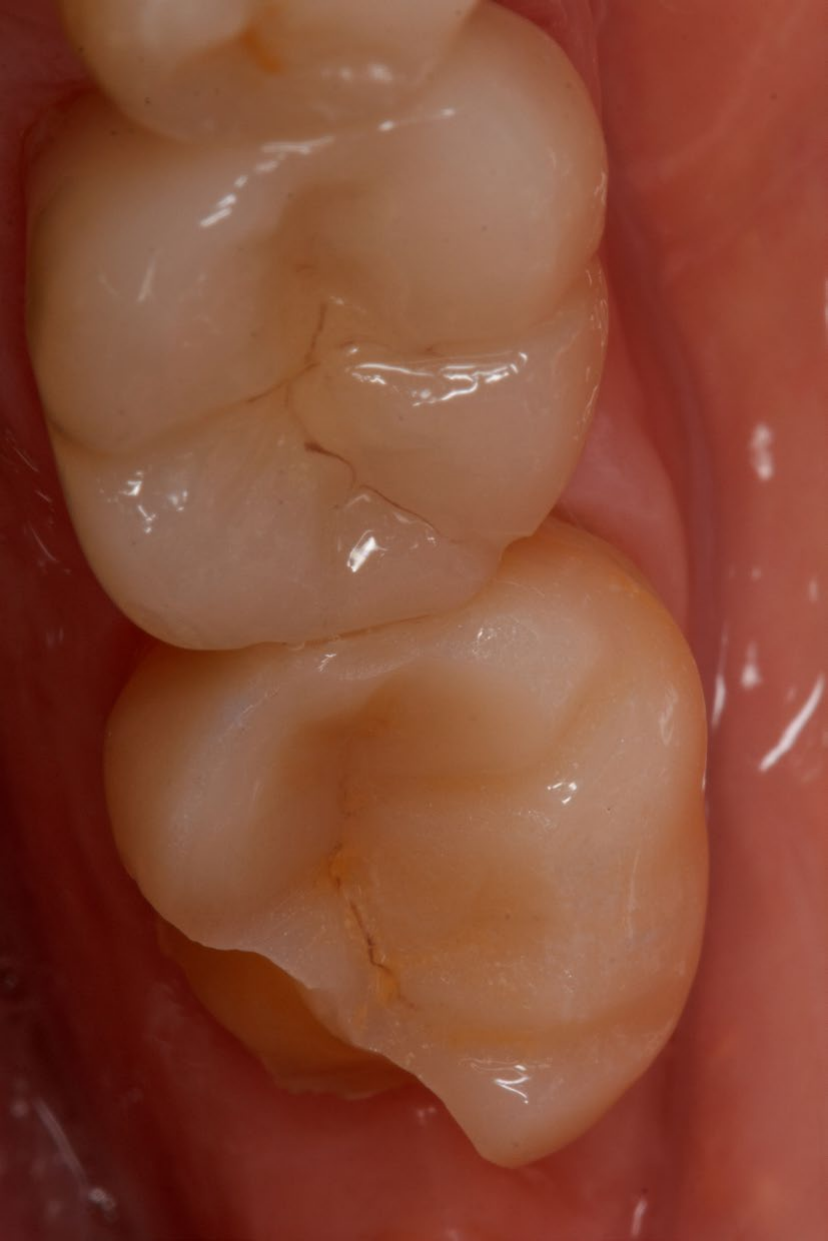
Complete failure due to a catastrophic fracture of the LZGC material at 38 months.

**FIGURE 5:**
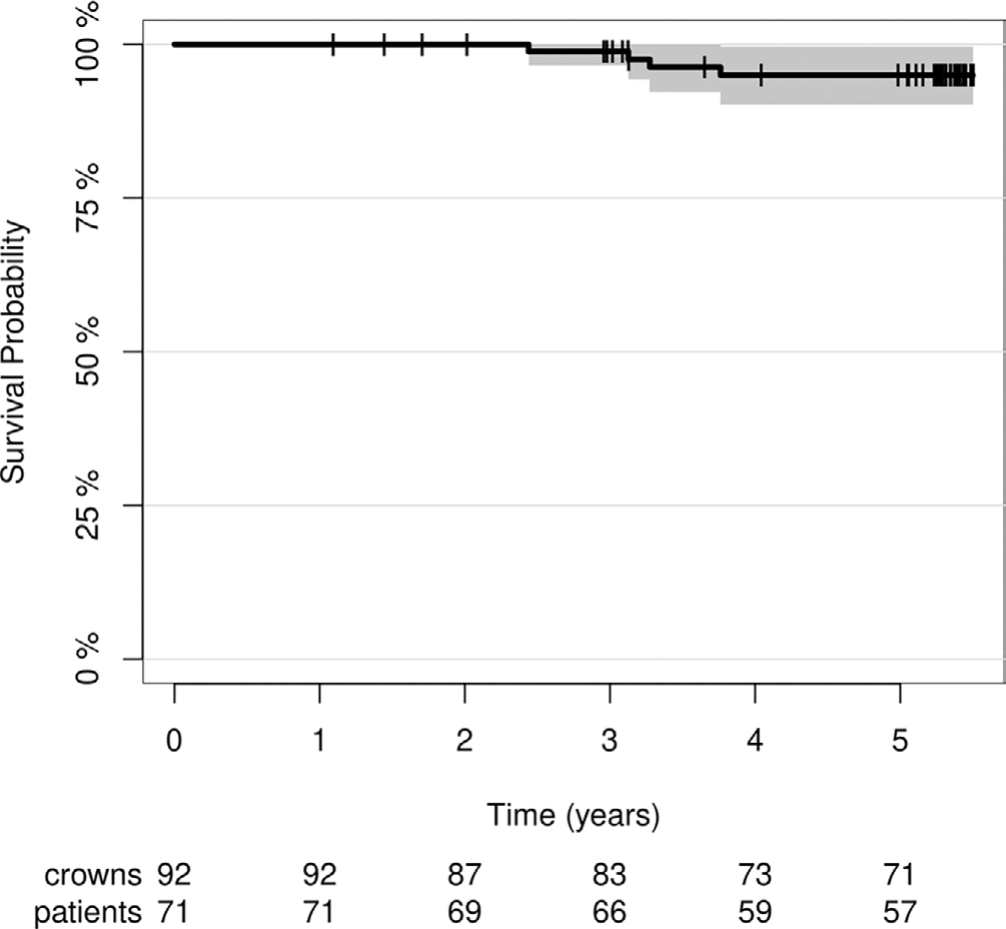
Overall survival rate of the CCPCs fabricated from an LZGC after a mean observational period of 5 years.

**FIGURE 6:**
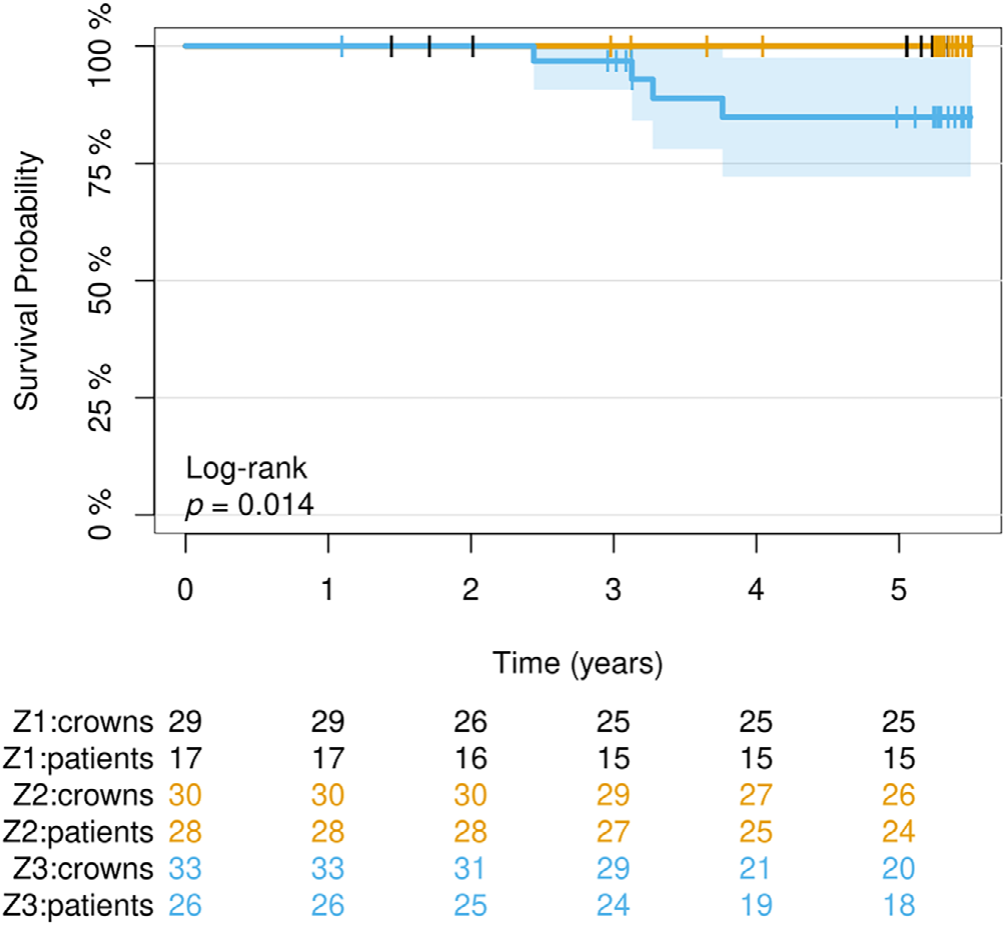
Survival probability of the CCPCs depending on the clinical setting/operator (centers 1–3).

At the 5‐year follow‐up examination, the loss of four CCPCs was documented. Two ceramic partial crowns in two different patients failed due to fractures of the abutment teeth (at 30 and 39 months), and another restoration in a different patient failed due to a catastrophic fracture of the ceramic (at 38 months) (Figure 4). One restoration had to be replaced following endodontic failure. Thus, 1 out of 4 complete failures was of technical origin, while the remaining losses were classified as biological failures. The overall survival rate after 5 years was 95% (95% confidence interval (95% CI): 0.9–1) (Figure 5). The 5‐year survival rate was 98% (95% CI: 0.93–1) for Group A and 93% (95% CI: 0.85–1) for Group B. The Cox proportional regression model showed no statistically significant difference in survival between the two groups (*p* = 0.347). The 5‐year survival rate for ceramic partial crowns placed on premolars was 100%, while the survival rate for those placed on molars was 94% (95% CI: 0.88–1). There was no significant difference between the survival curves for the different locations (premolar vs. molar) at the *p* < 0.05 level (*p* = 0.29). All complete failures occurred in one of the three centers (C3). The center‐specific survival rates were 100% for centers C1 and C2, while the survival rate for restorations placed in Center C3 was 85% (95% CI: 0.72–0.97). This difference was statistically significant (*p* = 0.014) (Figure 6). Therefore, the null hypothesis was partially rejected for the survival rate.

### 3.3 Success Rate

**FIGURE 7:**
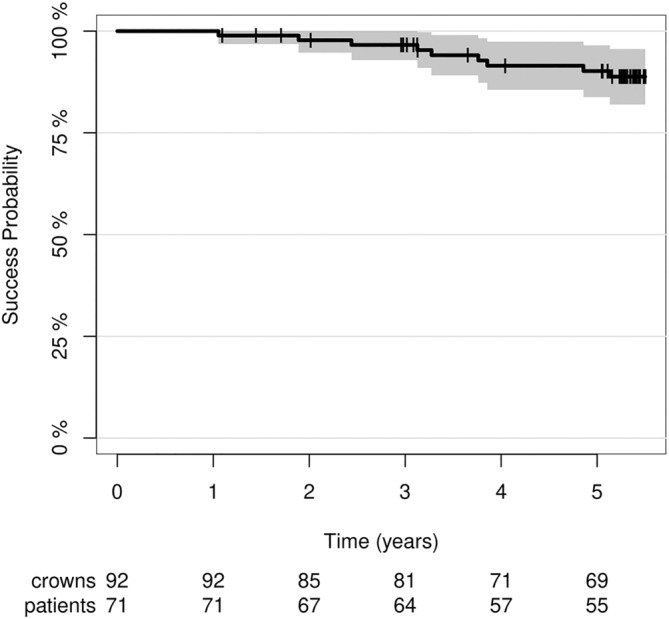
Overall success rate of the CCPCs fabricated from an LZGC after a mean observational period of 5 years.

**FIGURE 8:**
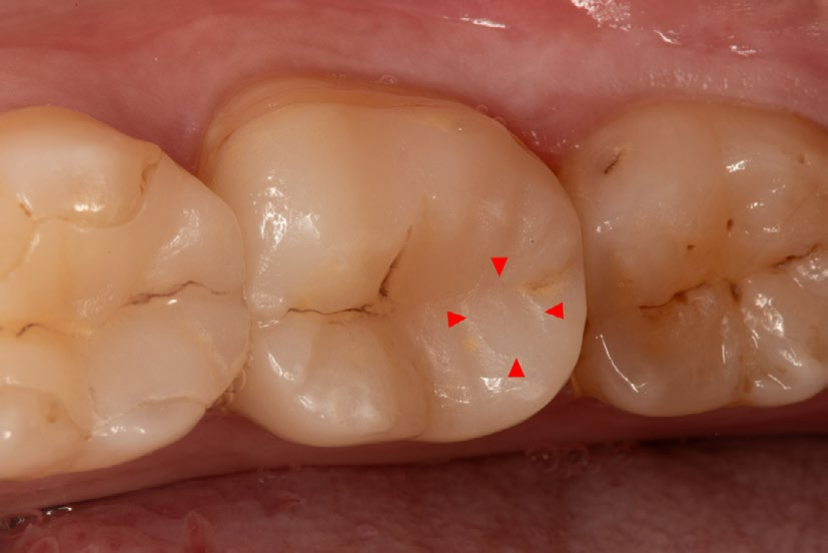
Minor fracture of the ceramic material that was polished to maintain function (fractured ceramic area is marked by red arrows).

In addition to the four complete failures, five more restorations required clinical intervention to maintain function. The time‐dependent total success rate (intervention‐free survival) was 90.0% (95% CI: 0.84–0.97) after 5 years (Figure 7). The clinical interventions required to keep the restorations functional were caused by two losses of vitality with subsequent endodontic treatments, one case of secondary caries requiring a composite restoration, and two minor ceramic fractures (< 2 mm^2^) that could be polished intraorally (Figure 8).

**FIGURE 9:**
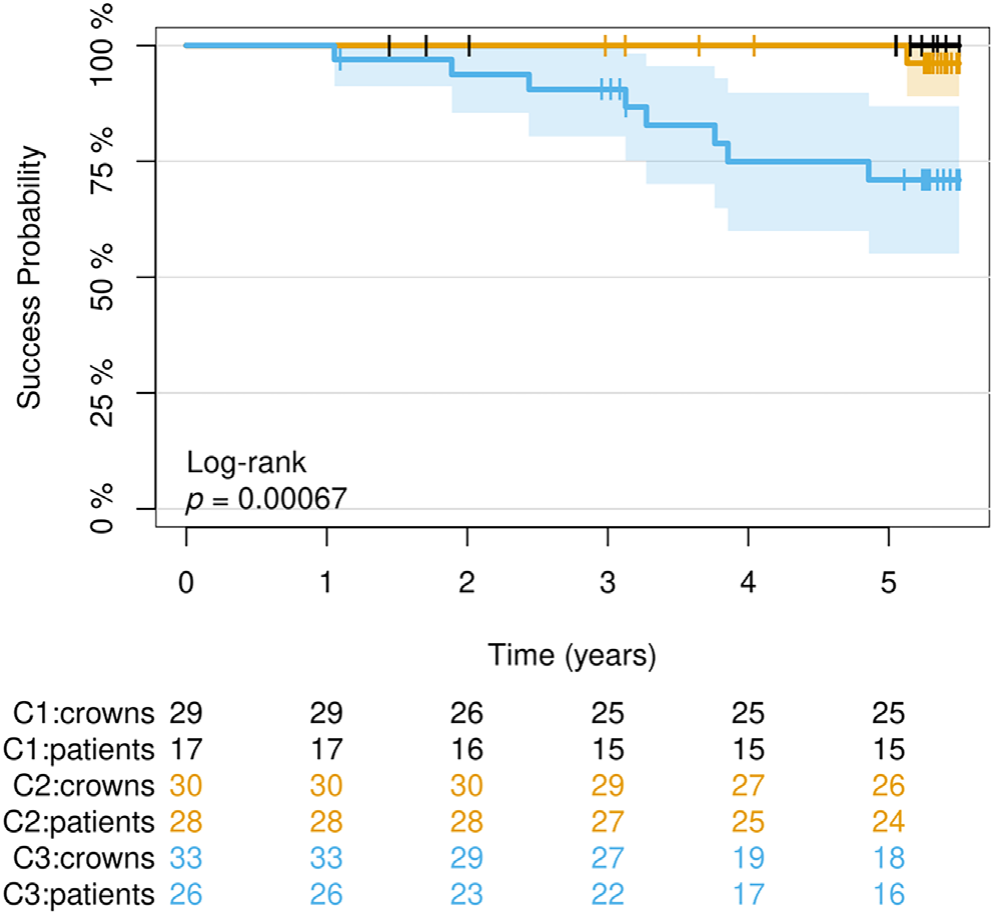
Success probability of the CCPCs based on the clinical setting/operator (Centers 1–3).

The 5‐year success rate for the CCPCs from Group A was 91% (95% CI: 0.82–0.99) and 90% (95% CI: 0.81–0.99) for the restorations from Group B. A total of 88% (95% CI: 0.74–1) of the CCPCs placed on premolars remained functional without intervention during the 5‐year observational period. The corresponding success rate for molar CCPCs was 91% (95% CI: 0.84–0.98). The success rate (intervention‐free survival) was unaffected by the position of the partial crowns (premolar vs. molar (hazard ratio (HR) = 0.979, *p* = 0.979)) and the cementation material (hazard ratio (HR) = 0.769, *p* = 0.685). All failures and interventions during the 5‐year follow‐up period occurred in one of the three centers, leading to a significant difference (log‐rank test: *p* = 0.00067) in the center‐specific 5‐year success rate (C1 + C2: 100%; C3: 71%; (95% CI): [0.55; 0.87]) (Figure 9). Restorations placed in C1 and C2 showed a significantly reduced risk for failure or clinical intervention (hazard ratio (HR) = 0.103, *p* = 0.026) compared with restorations placed in C3. The null hypothesis regarding the success rate was partially rejected.

At baseline, the marginal adaptation was rated alpha in 46 restorations (95.7%) in Group A and in 43 restorations (97.7%) in Group B. All other restorations were rated bravo for the criteria of marginal adaptation and marginal discoloration. At the 5‐year recall, alpha ratings in Group A decreased to 45.7% and 47.1% in Group B with the remaining restorations being rated bravo for both criteria (marginal adaptation and marginal discoloration) (Table 2). The marginal discoloration was rated alpha for all restorations in both groups at baseline. At the 5‐year clinical examination, the alpha ratings decreased to 57.1% (Group A) and 55.9% (Group B). Regarding the parameters “marginal adaptation” and “marginal discoloration,” a statistically significant difference (*p* < 0.05) could be found in both groups for the data assessed at baseline and 5 years (Table 2). Regarding the parameters “marginal adaptation” and “marginal discoloration,” no significant differences for the data assessed at the 5‐year recall were detected between the restorations in Group A and Group B.

**TABLE 2:** Rating for selected modified USPHS criteria [12] at the 5‐year clinical examination.

		Marginal adaptation			Marginal discoloration			
Group	Time	A	B	C	D	A	B	C
A	Baseline (*n* = 48)	46^c^ (95.7%)	2^c^ (4.3%)	0	0	48^c^ (100%)	0^c^	0
	5‐year (*n* = 35), 1170^a^	16^c^ (45.7%)	19^c^ (54.3%)	0	0	20^c^ (57.1%)	15^c^ (42.9%)	0
B	Baseline (*n* = 44)	43^c^ (97.7%)	1^c^ (2.3%)	0	0	44^c^ (100%)	0^c^	0
	5‐year (*n* = 34)^b^	16^c^ (47.1%)	18^c^ (52.9%)	0	0	19^c^ (55.9%)	15^c^ (44.1%)	0

^a^ Only 35 patients were included because one partial ceramic crown failed.

^b^ Only 34 patients were included because three partial ceramic crowns failed.

^c^ Significant difference between baseline and 5‐year investigation (*p* ≤ 0.05).

## 4 Discussion

In the present practice‐based study, lithia‐zirconia glass–ceramic CCPCs showed an overall 5‐year survival rate of 95%. The success rate was determined at 90%. This finding is in good accordance with the results reported from a retrospective trial for posterior full‐coverage monolithic lithium disilicate crowns fabricated with the Cerec system [18]. This university‐based study used the same survival and success criteria as the present study and reported a 6‐year cumulative survival rate of 93% and a success rate of 86.45%.

In the present study, 3 out of 4 complete failures were classified as biological failures (two tooth fractures, one endodontic failure), whereas only one failure was related to the restorative material (material fracture). This finding is in clear contrast to earlier studies evaluating CCPCs made from a feldspathic ceramic material. Based on the available clinical data for this type of material, a restoration‐fracture‐related failure rate of 5%–6% can be anticipated for the first 3 years of clinical service [12, 25, 29]. For the LZGC material applied in this study, after 5 years a fracture‐related failure rate of 1.3% was calculated. The lower material fracture rates can be attributed to the improved mechanical properties of the LZGCs used in the present study compared to the feldspathic porcelain. This effect has already been reported for other high‐strength glass–ceramics (i.e., lithium disilicate). In a systematic review evaluating the clinical performance of CAD/CAM‐fabricated lithium disilicate crowns (six studies: 154 patients with 204 crowns), biological complications occurred more often than technical complications [19].

In the present study, the survival and success rates were significantly affected by the clinical settings in which the restorations were placed (center effect). All restorations were fabricated using the same CAD/CAM system (Cerec system with Omnicam AC and Cerec MCXL milling) and the same type of ceramic material with a standardized clinical protocol for cementation. However, the operators' experience with the Cerec system differed for the three dentists involved in the clinical work. Two dentists (centers 1 and 2) had more than 10 years of clinical experience in the fabrication of chairside restorations with the Cerec system. The clinical experience of the third dentist was limited to 3 years. Interestingly, the restoration placed by dentists with prolonged clinical experience for chairside‐fabricated restorations (centers 1 and 2) resulted in 5‐year survival and success rates of 100%.

This result was consistent with the survival and success rates reported for conventionally fabricated posterior partial crowns placed by an experienced operator. In this practice‐based study on 765 adhesively luted lithium disilicate posterior partial crowns, a 5‐year survival rate of 99.6% and a success rate of 98.6% were reported [17]. On the other hand, these survival and success rates were significantly different from the survival (85%) and success (71%) rates determined in Center 3. The risk for a complete failure or a clinical intervention was 9.7‐fold higher for restorations placed in Center 3 than for the restorations placed in the other two centers. Variations in clinical experience might provide a possible explanation. In the specific setting of the present study, the general clinical experience and the experience with the Cerec system of the operator in Center 3 were limited (3 years). Therefore, the operator effect might not be limited to the level of experience with the Cerec system but also related to the level of general clinical experience.

Clinical data regarding the effect of the operator on the clinical performance of CAD/CAM‐fabricated crowns or partial crowns are still sparse [30]. In the university‐based study identified on this topic, the restorations were placed by faculty members and undergraduate students. In this setting, the operator experience did not affect the short‐term clinical performance of CCPCs [30]. For the interpretation of these results, it has to be considered that all clinical procedures performed by the students were supervised and controlled by graduate dentists. This quality assurance measure might diminish a potential operator effect. For conventionally fabricated indirect ceramic restorations, the operator effect was previously documented. In a 2‐center prospective practice‐based clinical trial with adhesively luted ceramic inlays, a significant operator‐dependent difference in the survival rates (operator A: 97.4% vs. operator B: 75.4%) at the same magnitude as noted in the present study was determined [31].

Possible sources of error directly related to the operators' general clinical experience are potentially noted in the preparation design, the amount of substance reduction, or the adhesive luting procedure [19, 23, 31]. For example, the need for endodontic treatment after cementation can be attributed to an overreduction of natural tooth structures or insufficient cooling procedure during preparation. On the other hand, an insufficient occlusal reduction leading to a violation of the minimum material thickness can cause material fracture [19]. The potential influence of the level of general clinical experience rather than the level of Cerec‐specific experience can be seen in the fact that 6 out of 9 complete failures or interventions were related to biological issues (tooth fractures, secondary caries, endodontic interventions). Based on the present study's findings, it appears that for chairside‐fabricated indirect restorations, a substantial difference in outcome between practitioners exists irrespective of the CAD/CAM systems or the material used. This finding strongly supports the need for adequate training for this type of restoration, as recommended for conventional indirect restorations [3].

Over the course of the present study, the restorations did not show any sign of debonding. This result aligns with other studies on the clinical performance of CCPCs bonded with dual‐curing composite cement using either a selective or total‐etch technique, which have reported debonding rates of 0%–3.6% over observational periods of 3–6 years [11, 12, 25]. Therefore, the results of this study confirm that using an adhesive luting procedure with a combination of the total‐etch or selective‐etch technique and dual‐curing cement reduces the risk of debonding for glass–ceramic CCPCs [11, 12, 25]. Furthermore, the results of the present study (no debonding) verify the good bonding properties of the ZLGC material that were already demonstrated in several in vitro studies under clinical conditions [22]. Additionally, the type of dual‐curing cement showed no statistically significant effect on clinical parameters, survival rates, or success rates. This result is consistent with a clinical study that compared the performance of glass–ceramic onlays luted with two different dual‐curing resin materials over three years [32]. In accordance with the present study, no differences between the two luting systems could be detected.

A systematic review of the longevity of ceramic onlays, encompassing 21 studies, found that marginal discolorations are a common issue, affecting 5.0%–88.2% of restorations. In the present study, a significant increase in bravo ratings was observed for both luting agents from the baseline to the 5‐year examination [9].

Regarding marginal adaptation over an observational period of 5 years, bravo ratings increased up to 50.6%–54.3%, which is statistically significant However, this result is well within the range reported for other evaluations with dual‐curing luting agents [8, 9, 11, 25]. Besides the cementation technique, the position of the restoration is considered a significant risk factor for all‐ceramic restorations. Higher occlusal forces lead to an increased failure risk for molar all‐ceramic restorations [9]. Several studies on feldspathic porcelain or leucite‐reinforced glass–ceramics have shown that molar restorations have higher failure rates. This indicates that the position of a restoration is indeed a relevant risk factor [7, 9, 10, 33].

To address the sensitivity of ceramic restorations, it is recommended to use high‐strength materials such as lithium disilicate ceramics or glaze‐fired lithia‐zirconia glass–ceramics [8, 19]. In this study, all failures occurred in CCPCs placed on molars, with a success rate of 94% (95% CI: 0.88–1), while all premolar restorations remained functional, achieving a 100% survival rate. The difference in time‐dependent survival was not statistically significant (*p* = 0.29). This finding is in accordance with the results of other clinical studies on posterior monolithic restorations made from high‐strength glass–ceramics (i.e., lithium disilicate) without detecting a significant difference in the survival rates depending on the position of the restoration [14, 15, 16]. Nevertheless, the findings of the present study have to be interpreted with caution, as a skewed distribution of restorations placed on premolars (*n* = 17) and molars (*n* = 56) was present. Moreover, only a limited number of events (*n* = 4) occurred during the 5‐year observational period. Therefore, it must be taken into account that the study was underpowered to detect this potential effect. Another limitation of the present study can be observed in the skewed distribution of restorations placed on vital (*n* = 68) and nonvital teeth (*n* = 5). The skewed distribution and the limited number of events rendered the evaluation of tooth vitality as a risk factor for clinical performance inconclusive. These factors represent the primary limitations of the present study. To better evaluate these risk factors, clinical studies with extended observational periods and larger sample sizes are required. Despite these limitations, this study provides valuable insights into the clinical assessment of chairside‐fabricated partial crowns. It provides 5‐year clinical results for relatively new LZGC materials, tested under real‐life conditions in three different private practices. To date, other studies have only been documented with shorter observational periods [26]. Another positive feature involves the multicenter design of the present study. Due to the combination of a sufficient and equal number of restorations placed by different operators with varying levels of specific experience with the Cerec system, an analysis of the operator as an influencing factor was possible. This effect has not been documented for chairside‐fabricated restorations to date.

Moreover, the importance of the data from the present study is supported by a comparison with the 3‐year data reporting on the same study population. At the 3‐year recall examination, only one complete failure and one clinical intervention to maintain function were reported. This resulted in a 3‐year survival rate of 99% and a 3‐year success rate of 98% [26]. During the 3–5‐year follow‐up, three more failures occurred, and four more interventions were required to maintain function. Thus, the survival rate decreased from 99% to 95%, and the success rate was reduced to 90%. These changes influenced the calculated annual failure rates. Based on the 3‐year results, an annual failure rate of 0.3% was calculated. This rate increased 3‐fold to 1% when calculated on the basis of the 5‐year clinical follow‐up results. This finding demonstrates the importance of clinical data generated from studies with prolonged observational periods to avoid an underestimation of the annual failure rate.

Taking the limitations into account, the results of this study can be seen as an initial clinical confirmation for the ZLGC material. Additionally, it supports the hypothesis of earlier in vitro studies that a decrease in fracture‐related failures compared to feldspathic porcelain is likely [1, 3, 6, 13, 20]. However, apart from the improved material properties and the usage of CAD/CAM fabrication technology, the operators represent a highly relevant influencing factor for the clinical performance of CCPCs fabricated under the typical conditions of private practice.

## 5 Conclusions

After a mean observational period of 5 years, biological reasons caused the majority of complete failures and interventions to maintain function for CCPCs made of an LZGC. The achievable survival and success rates were significantly influenced by the operator, whereas other risk factors (position of the restoration, type of the luting agent) demonstrated no association with clinical survival and success. More clinical long‐term data from practice‐based populations are needed for a more detailed evaluation of the detected effects.

## Conflicts of Interest

The authors declare no conflicts of interest.

## Data Availability

The data that support the findings of this study are available on request from the corresponding author. The data are not publicly available due to privacy or ethical restrictions.
